# Habitat modification by invasive crayfish can facilitate its growth through enhanced food accessibility

**DOI:** 10.1186/s12898-017-0147-7

**Published:** 2017-12-12

**Authors:** Shota Nishijima, Chisato Nishikawa, Tadashi Miyashita

**Affiliations:** 1 Laboratory of Biodiversity Science, School of Agricultural and Life Sciences, The University of Tokyo, 1-1-1 Yayoi, Bunkyo, Tokyo, 113-8657 Japan; 2 Present Address: National Research Institute of Fisheries Science, Japan Fisheries Research and Education Agency, 2-12-4 Fukuura, Kanazawa, Yokohama 236-8648 Japan

**Keywords:** Interaction modification, Invasive engineer, Macrophyte refuge, Positive density dependence, Red swamp crayfish, Submerged plants

## Abstract

**Background:**

Invasive ecosystem engineers can facilitate their invasions by modifying the physical environment to improve their own performance, but this positive feedback process has rarely been tested empirically except in sessile organisms. The invasive crayfish *Procambarus clarkii* is an ecosystem engineer that destroys aquatic macrophytes, which provide a physical refuge for animal prey, and this destruction is likely to enhance vulnerability to predators. Using two series of mesocosm experiments, we tested the hypothesis that the invasive crayfish increases its feeding efficiency on animal prey by reducing submerged macrophytes, thus increasing its individual growth rate in a positive density-dependent manner.

**Results:**

In the first experiment, increasing crayfish density reduced both macrophytes and animal prey (dragonfly and chironomid larvae) and, importantly, increased the growth rate of individual crayfish, in accordance with our expectation. In the second experiment, we used artificial macrophytes to clarify whether the physical architecture of macrophytes itself protects animal prey and limits crayfish growth rate. Increasing the artificial macrophyte quantity not only increased the survival of animal prey, but also retarded the crayfish growth rate.

**Conclusions:**

We conclude that macrophytes strengthen bottom-up control of crayfish, but this effect can be relaxed by increasing the density of crayfish via reduction in macrophytes. This positive feedback process may explain the crayfish outbreaks and regime shifts occasionally observed in invaded freshwater ecosystems.

**Electronic supplementary material:**

The online version of this article (10.1186/s12898-017-0147-7) contains supplementary material, which is available to authorized users.

## Background

Invasive species pose serious threats to biodiversity, food-web structures, biogeochemical processes, and physical habitat structures of non-native ecosystems [1]. In particular, when invaders are ecosystem engineers, which modify also their physical environment [2], they can exert profound impacts on their non-native ecosystems [3, 4]. One of the reasons for this is that environmental modification by engineers occasionally creates positive feedback that promotes their own population growth [5, 6] and thus invasion ability [7, 8]. Theoretically, positive feedback induced by ecosystem engineers can create alternative stable states in ecosystems, potentially causing catastrophic regime shifts [5, 9]. However, empirical evidence that ecosystem engineering actually improves the performance of the engineer is limited to sessile organisms, such as plants [10–12] and intertidal sessile invertebrates [13, 14].

Invasive crayfish such as *Procambarus clarkii* can abruptly become overabundant and then dramatically decrease the abundance and diversity of aquatic plants and animals in freshwater ecosystems [15–17]. Crayfish can be regarded as allogenic ecosystem engineers [2] because they modify physical habitats by burrowing activities and removing macrophytes that provide refuges for aquatic animals [18–20]. Since the physical architecture of macrophytes controls prey accessibility [21], the reduction of macrophyte refuges by crayfish (i.e., ecosystem engineering) elevates the crayfish predation rate on animal prey [18] and this can be considered a sort of “interaction modification”. This ecosystem engineering may further affect the crayfish itself; since a high predation rate implies a high foraging efficiency on animal prey, invasive crayfish may exhibit accelerated growth by the ecosystem-engineering effect. This “self-reinforcing” bottom-up effect, or positive feedback process, may be a mechanism underlying crayfish outbreaks in lakes and ponds, but it is not clear how changes in macrophyte and crayfish densities influence the growth rate of crayfish via changes in prey accessibility.

To address this question, we conducted two series of mesocosm experiments. In the first experiment, we examined how changes in crayfish density affect the individual growth rate of crayfish via changes in macrophyte density and prey capture rate. We predicted a positive density-dependence of crayfish growth. In the second experiment, we used artificial macrophytes to evaluate whether the physical structure of macrophytes affects prey catchability and consequently crayfish growth rate. We predicted that increasing the amount of artificial macrophytes would decrease the individual growth rate of crayfish owing to reduced animal prey availability.

## Methods

### Experiment I: varying crayfish density

We conducted the first experiment for 91 days from June to September of 2012 in the field at the University of Tokyo Tanashi Forest. We collected individual red swamp crayfish (*P. clarkii*) from Saitama Prefecture, central Japan. We used larvae of a native dragonfly (*Sympetrum baccha matutinum*) as animal prey for crayfish for the following three reasons. First, the abundance of dragonflies is known to decrease following crayfish outbreaks in ponds [16, 22]. Second, dragonfly larvae are an important food item for crayfish because they are large and easy to catch [19]. Third, dragonfly larvae utilize macrophytes as a refuge against predation from crayfish [18]. *S. baccha matutinum* used in the experiment is a common species distributed throughout Japan. We collected dragonfly individuals from Chiba prefecture, central Japan.

Chironomid larvae were used as animal prey for crayfish and dragonfly larvae. Chironomid larvae are commonly observed even when the abundance of dragonfly larvae is low due to a high crayfish density (S. Nishijima, unpublished). It appears that chironomid larvae are major animal prey, sustaining a high density of crayfish after other prey become scarce.

We used two species of submerged macrophytes: *Egeria densa* and *Elodea nuttallii*. Both species are not native to Japan, but are currently distributed widely throughout freshwater systems in Japan, allowing us to collect sufficient amounts of submerged macrophytes for the experiment. *E. densa* and *E. nutallii* were collected in, respectively, Tokyo and Ibaraki Prefectures, central Japan.

We used 20 mesocosm containers, each of which was 0.73 × 1.03 m^2^ with a depth of 0.3 m. The containers were allocated into seven different treatments (Table 1). Four treatments included macrophytes with various densities of crayfish (0, 1, 2 and 4 individuals per container). The treatments with crayfish had four replicates, and that with no crayfish had two replicates. The remaining three (with two replicates) are the control containers that did not contain macrophytes and had various crayfish densities (1, 2 and 4 individuals per container). The reason for the smaller number of replicates for the control treatments is that we intended to test the effect of crayfish density on crayfish growth, while the effects of the presence of macrophytes and crayfish on dragonfly larvae and macrophytes were already investigated in our previous study [18]. We also did not set the control treatment with no crayfish and no macrophytes, because almost all individuals of dragonfly larvae can survive in the absence of crayfish, regardless of the presence or absence of macrophytes [18].

**Table 1:** Experimental design. Shown are the number of replicates, experimental duration, and the number of introduced individuals, and measurement timing of prey items in the experiments I and II

Experiment I				
Number of replicates	Number of crayfish			
	0	1	2	4
w/ macrophytes	2	4	4	4
w/o macrophytes	–	2	2	2
Duration	91 days			
Dragonfly larvae	Introducing 34 individuals at the beginning and counting survivors on the 21th day			
Chironomid larvae	Introducing 30 individuals at the beginning and once a week and counting survivors at the end			

Experiment II			
Number of replicates	Artificial macrophyte density		
	No	Medium	High
w/ crayfish and dragonflies	4	4	4
w/o crayfish w/ dragonflies	4	–	–
w/ crayfish w/o dragonflies	4	–	–
Duration	28 days		
Dragonfly larvae	Introducing 50 individuals at first and then 20 individuals once a week and counting survivors twice a week		
Chironomid larvae	Introducing 30 individuals at the beginning and once a week and counting survivors at the end		

All crayfish individuals used were females, as they are more closely related to population growth in comparison with males. We used juvenile individuals with the initial wet weight of 5.5 ± 1.7 g (mean ± SD), and allocated them into each mesocosm so that initial weights in mesocosms became similar (one-way ANOVA: *F*_8,8_ = 0.55, *P* = 0.79). The abundance and biomass of crayfish in our experiment were slightly lower than those reported in the fields [15, 17, 23, 24] to simulate situations prior to crayfish overabundance. In the treatments with macrophytes, *E. densa* with 140 stems (wet mass ± SD: 280.0 ± 77.3 g) was collocated on one half of each container, while *E. nutalli* with 210 stems (157.1 ± 38.8 g) was collocated on the other half. Since *E. nutalli* have thinner stems than *E. densa*, the numbers of stems used for each mesocosm were different between macrophyte species. In all of the treatments, 34 individual dragonfly larvae were introduced to each mesocosm at the beginning of the experiment, and 30 individual chironomid larvae were added to each container once a week (Table 1). This is because ponds and lakes are open systems where aquatic insects, including chironomids, are intermittingly supplied; moreover, crayfish can walk around broader areas to search for food resources in real ecosystems than in our mesocosms. Ideally, dragonfly larvae should also have been introduced once a week, but as it was not possible to obtain sufficiently a large number of individuals from the fields, unlike commercially available chironoimds. Leaf litter of *Quercus serrata* and *Zelcova serrata*, collected from deciduous broad-leaved forests in the University of Tokyo Tanashi Forest, was used as a bottom substrate in containers (dry mass of 150 g for each container). Eight plastic pipes were added as shelters into each container to reduce crayfish cannibalism. The water depth was kept at about 15 cm for all containers during the experiment. Each container was covered by a wire mesh to prevent birds and mammals from accessing the setup.

To evaluate the amount of macrophytes, we estimated the coverage of macrophytes in water, rather than macrophyte biomass, to avoid potential disturbance caused by retrieving them from the container. We obtained four vertically aligned images (two for the side with *E. densa* and two for the side with *E. nutallii*) of the water in each container once or twice a week. We then quantified the proportional area covered by macrophytes using Image J (http://rsb.info.nih.gov/ij/) and obtained average estimates for the four images for each container. We measured the wet weight of each crayfish individual once a week to calculate its growth rate. We counted the numbers of dragonfly and chironomid larvae only once to avoid large disturbances; dragonfly individuals were counted on the 21th day after the beginning of the experiment (before they started to emerge to adults) and chironomid individuals at the end of experiment.

We performed three types of statistical analysis using generalized linear models (GLMs). First, we tested the effects of crayfish density (i.e., independent variable) on the numbers of surviving dragonfly and chironomid larvae (dependent variables), using only the treatments with crayfish. To determine appropriate error distributions, we conducted a likelihood ratio test by comparing likelihoods of models with Poisson and negative binomial distributions. As a result, a Poisson distribution was used for dragonfly larvae, since the null hypothesis of a Poisson distribution was not rejected (*χ* ^2^ = 0.25, *P* = 0.31). However, a negative binomial distribution was used for chironomid larvae, since the null hypothesis was rejected (*χ* ^2^ = 4.28, *P* = 0.019). We used the log-link function for both analyses.

In the second analysis, the effects of crayfish on macrophytes were tested. We used macrophyte coverage obtained on the 40th day after the beginning of the experiment as the dependent variable. This time period was selected because macrophytes in some containers completely disappeared thereafter. We used the logarithmic macrophyte coverages at the beginning of the experiment as an offset term to analyze the change rate of macrophytes, and the number of crayfish individuals as the independent variable. Here we did not include the treatments with no crayfish, as they had only two replicates. We employed a gamma distribution for the error term with the log-link function because the assumption of normality was not satisfied.

Lastly, we investigated the effect of crayfish density on the growth rate of crayfish themselves. We used the log-response ratio of the final to initial body weights, averaged across all individuals in a container, as the dependent variable:

$$\frac{1}{n}\mathop \sum \limits_{i = 1}^{n} \log \left[ {\frac{{Final \, weight_{i} }}{{Initial \, weight_{i} }}} \right],$$

where *n* is the number of crayfish individuals in each container and the subscript *i* represents each crayfish individual. We excluded from the analysis the cases where a dead body was heavily injured by other crayfish (*N* = 2) because surviving individuals would gain excessive weight by cannibalism. We assumed that the error term followed a normal distribution, which was confirmed by the Shapiro–Wilk normality test (*W* = 0.93, *P* = 0.45).

We determined the statistical significance of each independent variable based on a likelihood ratio test. Using the parametric bootstrap method [25], we tested whether the difference in the deviance (*D* = − 2*LL*, where *LL* is the log-likelihood) between the full model (including the independent variable) and the reduced model (excluding the independent variable from the full model) is significant. We first resampled data by generating random numbers from the error distribution under the assumption that the reduced model is correct. We then fitted both full and reduced models to each resampled data set and calculated the difference in the deviance between both models. We lastly obtained *P* value, or the probability that the difference in the deviance for the resampled data exceeded the observed one. We generated 10,000 resampled data sets in the bootstrap. As the bootstrap method may not work well for small sample sizes [25], we also performed a model selection approach based on AICc (a small sample version of AIC) [25]. These statistical analyses were performed using the statistical software R [26] with the packages “glmmADMB” [27, 28], “MASS” [29], and “pscl” [30].

### Experiment II: varying artificial macrophyte density

In the second mesocosm experiment, we tested if increasing the abundance of artificial macrophytes decreases the foraging efficiency and individual growth rate of crayfish. This experiment was performed for 28 days from June to July of 2011 in the field at the University of Tokyo Tanashi Forest. We collected crayfish, dragonfly larvae (*S. baccha matutinum*), and chironomid larvae from the same locations as those in the first experiment. We used commercially available plastic macrophytes that crayfish cannot cut and hence cannot reduce. These artificial macrophytes were made to mimic the submerged plant *Limnophila sessiliflora*, which is distributed widely in farm ponds, paddy fields, and water channels in Asian countries, including Japan [31]. Owing to relatively high structural complexity, we expected this species to have an important refuge function for dragonfly and chironomid larvae. One set of artificial macrophytes had four stems of about 10 cm in height.

We allocated 20 mesocosm containers (the same as in the first experiment) equally into five treatments (i.e., four replicates per treatment; Table 1). Three out of the five treatments involved crayfish, dragonfly, and chironomid larvae, with different densities of artificial macrophytes (zero: 0 sets, medium: 72 sets, high: 144 sets), which were determined based on natural densities of *L. sessiliflora* in farm ponds [32]. Another treatment involved prey but no crayfish as a control to clarify the top-down effect of crayfish on prey. The other involved crayfish and chironomid larvae, but no dragonfly larvae to reveal their bottom-up effect; we expected dragonflies to contribute substantially to crayfish growth. These two control treatments did not include artificial macrophytes.

We introduced three female crayfish individuals into each mesocosm. To measure growth in body weights, we used small-sized individuals with a wet mass of 2.1 ± 0.5 g. The initial weights of crayfish were not different among mesocosms (one-way ANOVA: *F*_7,16_ = 0.004, *P* = 1.00). We introduced 50 dragonfly larvae at the beginning of the experiment, and then added 20 individuals once per week (Table 1). More dragonfly larvae were used in this experiment than in the first experiment is because the artificial material cannot be consumed by crayfish. We counted the number of surviving dragonfly larvae twice a week. Furthermore, we added 30 chironomid larvae at the start and thereafter once per week. We examined the number of surviving chironomid larvae at the end of the experiment. The densities of dragonfly and chironomid larvae in the experimental setting were within the ranges of natural densities [22, 23, 33]. Leaf litter with dry mass of 300 g was introduced into each container as a bottom substrate. Other experimental settings (plastic pipes, water depth, and wire mesh) were the same as in the first experiment.

We performed two types of statistical analyses using GLMs. We first analyzed the number of surviving dragonfly and chironomid larvae at the end of experiment as dependent variables. Independent variables were the presence or absence of crayfish and the density of artificial macrophytes, which was treated as a continuous variable (the same applies hereafter). We used a negative binomial distribution for the error term in the dragonfly analysis, because the null hypothesis of a Poisson distribution was rejected (*χ* ^2^ = 2.73, *P* = 0.049). On the other hand, we assumed a Poisson distribution for chironomid larvae, because the null hypothesis of a Poisson distribution was not rejected (*χ* ^2^ = 0.00, *P* = 0.500). We used the log-link function for both analyses.

The second analysis used the average growth rate of three crayfish individuals in a mesocosm as a dependent variable. Individual growth rates during the experiment were expressed as the log-response ratio of the final to initial body weights. In a case where a dead individual was replaced with a fresh one, we summed the growth rates of the original individual (from the start to death) and the second individual (from introduction to finish). When a dead body injured by other crayfish was found, however, these cases were excluded from the statistical analysis (*N* = 4), as in the first experiment. Independent variables were the presence or absence of dragonfly larvae and the artificial macrophyte density. Here a normal distribution was used because the Shapiro–Wilk normality test supported the normality assumption (*W* = 0.96, *P* = 0.74).

For both kinds of analyses, we conducted a likelihood ratio test based on the parametric bootstrap method. We calculated the difference in the deviance between the full model and the reduced model. The other processes are the same as in the first experiment. We also performed a model selection approach based on AICc. The statistical analyses were performed using the statistical software R [26] with the packages “glmmADMB” [27, 28], “MASS” [29], and “pscl” [30].

## Results

### Experiment I: varying crayfish density

**Fig. 1:**
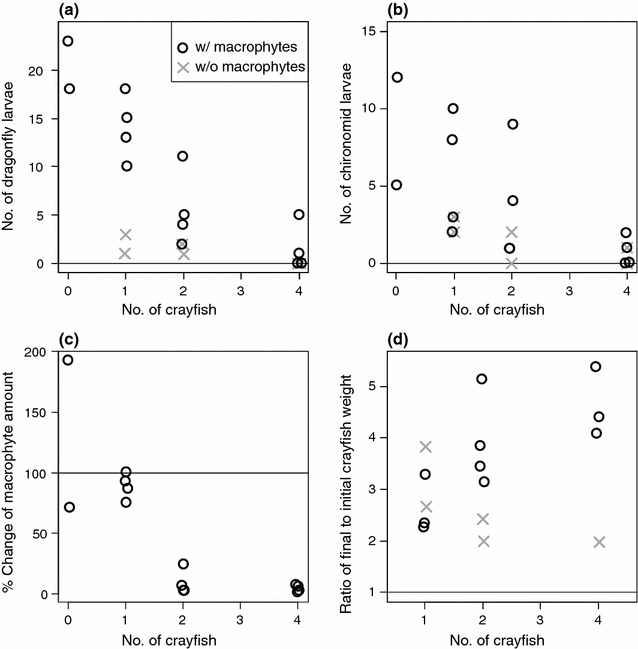
Results of experiment I: **a** the number of surviving dragonfly larvae on the 21th day after the beginning of the experiment, **b** the number of surviving chironomid larvae at the end of the experiment, **c** the change of macrophyte abundances during the first 40 days of the experiment, and **d** the individual crayfish growth rate during the experiment (ratio of final to initial crayfish weight). The black “O” and the gray “X” represent the presence and absence, respectively, of macrophytes

An increased crayfish density significantly reduced the numbers of dragonfly larvae (*ΔD* (difference in deviance between models) = 48.03, *P* < 0.0001; Fig. 1a) and chironomid larvae (*ΔD* = 7.02, *P* = 0.016; Fig. 1b). Furthermore, an increased crayfish density significantly decreased the macrophyte abundances (*ΔD* = 14.78, *P* = 0.001; Fig. 1c). When there was a single crayfish individual in a container, the amount of macrophytes did not change substantially and remained at a level similar to that in the case without crayfish.

The crayfish growth rate increased significantly as the number of crayfish increased (i.e., positive density-dependence) in the presence of macrophytes (*ΔD* = 7.867, *P* = 0.016; Fig. 1d). In contrast, crayfish seemed to exhibit negative density-dependence in the absence of macrophytes (Fig. 1d). The model selection based on AICc supported the results of the likelihood ratio tests, because the models including the crayfish density were selected as the best models with high Akaike weights (*w* > 0.8; Additional file 1: Table S1).

### Experiment II: varying artificial macrophyte density

**Fig. 2:**
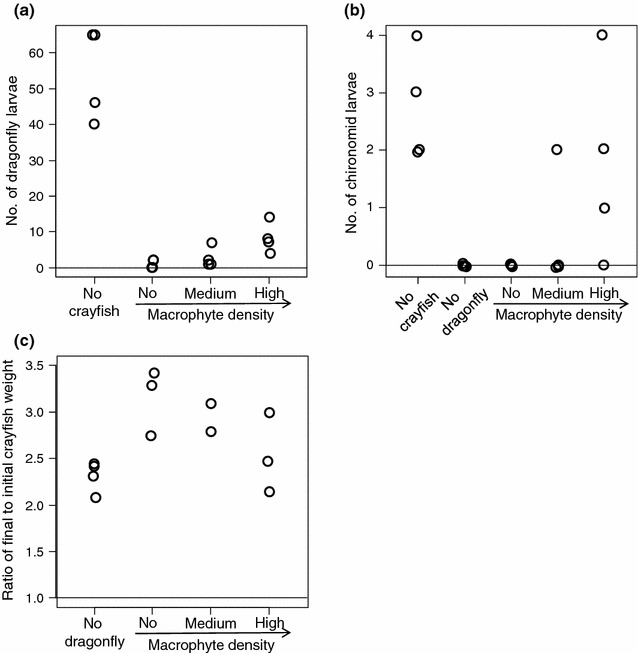
Results of experiment II: **a** the number of surviving dragonfly larvae at the end of the experiment, **b** the number of surviving chironomid larvae at the end of the experiment, and **c** the individual crayfish growth rate during the experiment (ratio of final to initial crayfish weight)

Crayfish significantly decreased the numbers of dragonfly larvae (*ΔD* = 33.82, *P* < 0.0001; Fig. 2a) and chironomid larvae (*ΔD* = 24.89, *P* < 10^−4^; Fig. 2b), consistent with the first experiment. An increase in artificial macrophyte density, however, significantly increased the numbers of surviving dragonfly larvae (*ΔD* = 17.42, *P* = 0.0004; Fig. 2a) and chironomid larvae (*ΔD* = 14.18, *P* < 0.0001; Fig. 2b).

Dragonfly larvae significantly promoted the crayfish growth rate (*ΔD* = 12.065, *P* < 0.0036; Fig. 2c). Then, an increase in artificial macrophyte density significantly affected the crayfish growth rate (*ΔD* = 6.231, *P* = 0.0305; Fig. 2c). It is noteworthy that the highest artificial macrophyte density suppressed the crayfish growth rate to a level close to that of the treatment without dragonflies (Fig. 2c). The model selection based on AICc supported the results of the likelihood ratio tests, because the models including the density of artificial macrophytes were selected as the best models with high Akaike weights (*w* > 0.6; Additional file 1: Table S2).

## Discussion

Based on a set of mesocosm experiments, we demonstrated that invasive crayfish can increase their own growth rate by habitat modification, or ecosystem engineering. In the first experiment, we demonstrated that increases in crayfish density increased the crayfish growth rate in the presence of submerged macrophytes (Fig. 1). In the second experiment, using artificial macrophytes, we demonstrated the physical function of macrophytes for crayfish-prey interactions. Increased macrophyte refuges not only enhanced the survival rates of animal prey, but also affected the crayfish growth rate (Fig. 2). Therefore, the positive density-dependence for crayfish growth in the first experiment can be attributed to the indirect effect of macrophyte reductions as refuges, leading to an increase in the feeding efficiency of crayfish on animal prey. To our knowledge, this is the first empirical evidence that a non-sessile ecosystem engineer facilitates its own performance through physical habitat modification. This is also an important demonstration that macrophytes control bottom-up limitations for predators in addition to top-down forces by predators. Whether this phenomenon can also occur in real ecosystems should be investigated in more natural settings in the future.

For positive density-dependence in crayfish growth to be feasible, the positive effect of high crayfish densities (i.e., elevated feeding rates on animal prey in response to a loss of macrophyte refuges) must exceed the negative effect (i.e., decreased per-capita resources). Our experiment showed that a decrease or loss of macrophytes increased the predation rate of crayfish on dragonfly larvae (Fig. 1a). This is consistent with the previous finding that submerged macrophytes have a protective effect on dragonfly larvae [18, 34]. In addition, more chironomid larvae were also preyed upon by crayfish when the density of macrophytes was low (Fig. 1b). We inferred that the increased prey accessibility in response to macrophyte reduction by conspecifics overrode the potential disadvantages of intraspecific competition for prey.

A major concern is whether invasive crayfish can maintain high densities in the long term. Even when increased foraging efficiency on animal prey temporarily increases the crayfish growth rate and thus density, reductions in the abundance of animal prey might eventually depress crayfish density. However, the animal prey we used (i.e., dragonfly and chironomid larvae) are widely distributed and allochthonously brought into ponds and lakes. In particular, chironomid larvae are commonly observed even when dragonfly larvae are rare owing to a high crayfish density (S. Nishijima, unpublished). Moreover, omnivorous crayfish exhibit moderate ontogenetic diet shifts from animal items to detritus with increasing size [35] and are supported by large quantities of allochthonous litter from surrounding forests in Japanese farm ponds [24]. We expect, therefore, that allochthonous inputs, such as wide-ranging aquatic invertebrates and leaf litter, contribute to the maintenance of high densities of invasive crayfish. Nevertheless, it is important to note that local populations of rare species with limited dispersal capacity are not rescued in the presence of high crayfish predation.

This study has implications for managing invasive crayfish and conserving macrophytes and aquatic invertebrates. Positive density-dependence in individual crayfish growth can be regarded as a component Allee effect [36]. Therefore, the successful invasion and spread of crayfish may be limited by the Allee effect and the population growth rate might be suppressed if intensive management of crayfish can reduce the density to a sufficiently low level. This could maintain populations of submerged macrophytes and dragonflies even in the presence of crayfish predation (Fig. 1a, c), if other major herbivores do not exist. A beneficial effect of crayfish fragmentation on macrophyte species that readily root adventitiously is also expected under low density conditions of invasive crayfish [37]. Biological control by predatory fish, combined with intensive trapping, seems effective for suppressing the invasive crayfish abundance to low levels [38, 39], yet is difficult or not feasible where native predatory fish are uncommon (e.g., Japan). As an alternative, introducing macrophytes having high tolerance to crayfish cutting and feeding within established exclosures can keep crayfish density at low levels, allowing coexistence of crayfish and aquatic invertebrates at small scales [40, 41]. In this context, more systematic studies are required to identify the macrophyte traits that determine the tolerance and vulnerability to crayfish cutting and feeding, although some studies already showed such a difference using a limited number of macrophyte species [17, 18]. Furthermore, restoration experiments on ponds and lakes are needed to test the feasibility and applicability of low-density management of invasive crayfish and establishing macrophytes within exclosures.

## Conclusions

Using two series of mesocosm experiments, we demonstrated that physical habitat modification by invasive crayfish causes positive density-dependence of the individual growth rate in the species. Theory suggests that positive feedback induced by ecosystem engineers can generate alternative states in ecosystems [5, 9]. Invasive crayfish sometimes reach high density and cause regime shifts from a clear-water state with abundant submerged plants to a turbid state with dominant phytoplankton [15, 20], suggesting the occurrence of alternative states. The mechanism underlying the positive density-dependence in our experiments may explain the alternative states and regime shifts in freshwater systems invaded by exotic crayfish. Once invasive alien species cause regime shifts, various management actions in addition to the direct control of invaders are often required for ecosystem restoration [42]. Such actions include the utilization of predatory fishes and reducing over-abundant leaf litter, an alternative resource for crayfish [24, 38, 43, 44]. Furthermore, introducing submerged plants that are tolerant to crayfish cutting and feeding may be effective for rebuilding macrophytes and aquatic invertebrates at small scales.

## Additional file

**Additional file 1: Table S1.** Model selection table of the experiment I. **Table S2.** Model selection table of the experiment II.

## References

[1] Davis MA (2009). Invasion biology.

[2] Jones CG, Lawton JH, Shachak M (1997). Positive and negative effects of organisms as physical ecosystem engineers. Ecology.

[3] Vitousek PM (1990). Biological invasions and ecosystem processes: towards an integration of population biology and ecosystem studies. Oikos.

[4] Lockwood JL, Hoopes MF, Marchetti MP (2008). Invasion ecology.

[5] Cuddington K, Wilson WG, Hastings A (2009). Ecosystem engineers: feedback and population dynamics. Am Nat.

[6] Wilson WG, Byers JE, Wilson WG, Hastings A, Cuddington K (2007). A new spirit and concept for ecosystem engineering. Ecosystem engineers: plants to protists.

[7] Cuddington K, Hastings A (2004). Invasive engineers. Ecol Modell.

[8] Gonzalez A, Lambert A, Ricciardi A (2008). When does ecosystem engineering cause invasion and species replacement?. Oikos.

[9] Nishijima S, Takimoto G, Miyashita T (2016). Autochthonous or allochthonous resources determine the characteristic population dynamics of ecosystem engineers and their impacts. Theor Ecol.

[10] Reinhart KO, Maestre FT, Callaway RM (2006). Facilitation and inhibition of seedlings of an invasive tree (*Acer platanoides*) by different tree species in a mountain ecosystem. Biol Invasions.

[11] Altieri AH, Bertness MD, Coverdale TC, Axelman EE, Herrmann NC, Szathmary PL (2013). Feedbacks underlie the resilience of salt marshes and rapid reversal of consumer-driven die-off. Ecology.

[12] Bertness MD, Yeh SM (1994). Cooperative and competitive interactions in the recruitment of marsh elders. Ecology.

[13] Bertness MD, Leonard GH (1997). The role of positive interactions in communities: lessons from intertidal habitats. Ecology.

[14] Lenihan HS (1999). Physical-biological coupling on oyster reefs: how habitat structure influences individual performance. Ecol Monogr.

[15] Rodríguez CF, Bécares E, Fernández-Aláez M (2003). Shift from clear to turbid phase in Lake Chozas (NW Spain) due to the introduction of American red swamp crayfish (*Procambarus clarkii*). Hydrobiologia.

[16] Maezono Y, Miyashita T (2004). Impact of exotic fish removal on native communities in farm ponds. Ecol Res.

[17] Gherardi F, Acquistapace P (2007). Invasive crayfish in Europe: the impact of *Procambarus clarkii* on the littoral community of a Mediterranean lake. Freshw Biol.

[18] Sato M, Nishijima S, Miyashita T (2014). Differences in refuge function for prey and tolerance to crayfish among macrophyte species. Limnology.

[19] Momot W (1995). Redefining the role of crayfish in aquatic ecosystems. Rev Fish Sci.

[20] Matsuzaki SS, Usio N, Takamura N, Washitani I (2009). Contrasting impacts of invasive engineers on freshwater ecosystems: an experiment and meta-analysis. Oecologia.

[21] Diggins MR, Summerfelt RC, Mnich MA (1979). Altered feeding electivity of the bluegill from increased prey accessibility following macrophyte removal. Proc Okla Acad Sci.

[22] Fukui M, Iwamoto A (2001). A study of the way to breed *Libellula angelina* in the Okegayanuma, Iwata-city (in Japanese). Insects Nat.

[23] Maezono Y, Kobayashi R, Kusahara M, Miyashita T (2005). Direct and indirect effects of exotic bass and bluegill on exotic and native organisms in farm ponds. Ecol Appl.

[24] Kobayashi R, Maezono Y, Miyashita T (2011). The importance of allochthonous litter input on the biomass of an alien crayfish in farm ponds. Popul Ecol.

[25] Burnham KP, Anderson DR (1998). Model selection and inference: a practical information-theoretic approach.

[26] R Core Team. R. A language and environment for statistical computing. Vienna: R Foundation for Statistical Computing; 2015. http://www.r-project.org/.

[27] Skaug H, Fournier D, Bolker B, Magnusson A, Nielsen A. Generalized linear mixed models using AD Model Builder. R package version 0.8.0; 2014.

[28] Fournier DA, Skaug HJ, Ancheta J, Ianelli J, Magnusson A, Maunder MN (2012). AD Model Builder: using automatic differentiation for statistical inference of highly parameterized complex nonlinear models. Optim Methods Softw.

[29] Venables WN, Ripley BD (2002). Modern applied statistics with S.

[30] Jackman S. pscl: classes and methods for R Developed in the Political Science Computational Laboratory, Stanford University. Department of Political Science, Stanford University. Stanford, California. R package version 1.4.9; 2015.

[31] Kadono Y (1994). Aquatic plants of Japan (in Japanese).

[32] Wang G-X, Watanabe H, Uchino A, Itoh K (2000). Response of a sulfonylurea (SU)-resistant biotype of *Limnophila sessiliflora* to selected SU and alternative herbicides. Pestic Biochem Physiol.

[33] Benke A (1976). Dragonfly production and prey turnover. Ecology.

[34] Rantala MJ, Ilmonen J, Koskimäki J, Suhonen J, Tynkkynen K (2004). The macrophyte, *Stratiotes aloides*, protects larvae of dragonfly *Aeshna viridis* against fish predation. Aquat Ecol.

[35] Correia AM, Anastacio PM (2008). Shifts in aquatic macroinvertebrate biodiversity associated with the presence and size of an alien crayfish. Ecol Res.

[36] Courchamp F, Berec L, Gascoigne J (2008). Allee effects in ecology and conservation.

[37] Lodge DM, Kershner MW, Aloi JE, Covich AP (1994). Effects of an omnivorous crayfish (*Orconectes rusticus*) on a freshwater littoral food web. Ecology.

[38] Hein CL, Roth BM, Ives AR, Vander Zanden MJ (2006). Fish predation and trapping for rusty crayfish (*Orconectes rusticus*) control: a whole-lake experiment. Can J Fish Aquat Sci.

[39] Gherardi F, Aquiloni L, Diéguez-Uribeondo J, Tricarico E (2011). Managing invasive cryfish: is there hope?. Aquat Sci.

[40] Doyle RD, Smart RM, Guest C, Bickel K (1997). Establishment of native aquatic plants for fish habitat: test plantings in two north Texas reservoirs. Lake Reserv Manag.

[41] Smart RM, Dick GO, Doyle RD (1998). Techniques for establishing native aquatic plants. J Aquat Plant Manag.

[42] Byers JE, Cuddington K, Jones CG, Talley TS, Hastings A, Lambrinos JG (2006). Using ecosystem engineers to restore ecological systems. Trends Ecol Evol.

[43] Miyake M, Miyashita T (2011). Identification of alien predators that should not be removed for controlling invasive crayfish threatening endangered odonates. Aquat Conserv Mar Freshw Ecosyst.

[44] Kennedy TA, Finlay JC, Hobbie SE (2005). Eradication of invasive *Tamarix ramosissima* along a desert stream increases native fish density. Ecol Appl.

