# Supplementary material for: Habitat modification by invasive crayfish can facilitate its growth through enhanced food accessibility
Source: BMC Ecol. 2017 Dec 12;17:37. doi: 10.1186/s12898-017-0147-7 (PMC5725987; doi:10.1186/s12898-017-0147-7)
Supplement: Supplementary file 1 — Additional file 1: Table S1. Model selection table of the experiment I. Table S2. Model selection table of the experiment II. [file 12898_2017_147_MOESM1_ESM.docx]

**Table S1.** Results of model selection in the experiment I. AICc, △AICc, the coefficients of variable (crayfish density) divided by standard errors, the number of parameters used (*k*), log-likelihoods (*LL*), and Akaike weights (*w*) are shown.

| AICc | △AICc | Crayfish | *k* | *LL* | *w* |
| --- | --- | --- | --- | --- | --- |
| Dragonfly larvae (*N* = 12) | |  |  |  |  |
| 64.19 | 0 | −5.66 | 2 | −29.43 | 1.00 |
| 109.29 | 45.10 | - | 1 | −53.44 | 0.00 |
|  |  |  |  |  |  |
| Chironomid larvae (*N* = 12) | |  |  |  |  |
| 58.58 | 0 | −2.85 | 3 | −24.79 | 0.84 |
| 61.93 | 3.35 | - | 2 | −28.30 | 0.16 |
|  |  |  |  |  |  |
| Macrophyte coverage (*N* = 12) | |  |  |  |  |
| −20.85 | 0 | −5.82 | 3 | 14.92 | 1.00 |
| −9.74 | 11.11 | - | 2 | 7.54 | 0.00 |
|  |  |  |  |  |  |
| Crayfish growth (*N* = 10) | |  |  |  |  |
| 4.94 | 0 | 3.09 | 3 | 2.53 | 0.86 |
| 8.52 | 3.58 | - | 2 | −1.40 | 0.14 |

**Table S2.** Results of model selection based in the experiment II. AICc, △AICc, the coefficients of variable (crayfish presence, dragonfly presence, and artificial macrophyte density) divided by standard errors, the number of parameters used (*k*), log-likelihoods (*LL*), and Akaike weights (*w*) are shown.

| AICc | △AICc | Crayfish | Dragonfly | Macrophyte | *k* | *LL* | *w* |
| --- | --- | --- | --- | --- | --- | --- | --- |
| Dragonfly larvae (*N* = 16) | |  |  |  |  |  |  |
| 92.27 | 0 | −9.50 | NA | 4.43 | 4 | −40.32 | 1.00 |
| 106.06 | 13.79 | −6.21 |  | - | 3 | −49.03 | 0.00 |
| 122.11 | 29.84 | - |  | - | 2 | −58.59 | 0.00 |
| 122.45 | 30.18 | - |  | −1.64 | 3 | −57.22 | 0.00 |
|  |  |  |  |  |  |  |  |
| Chironomid larvae (*N* = 20) | |  |  |  |  |  |  |
| 43.27 | 0 | −3.23 | NA | 2.77 | 3 | −17.82 | 1.00 |
| 54.65 | 11.38 | −3.53 |  | - | 2 | −24.97 | 0.00 |
| 64.06 | 20.80 | - |  | - | 1 | −30.92 | 0.00 |
| 65.37 | 22.10 | - |  | 1.11 | 2 | −30.33 | 0.00 |
|  |  |  |  |  |  |  |  |
| Crayfish growth (*N* = 12) | |  |  |  |  |  |  |
| −8.79 | 0 | NA | 3.95 | −2.48 | 4 | 11.25 | 0.61 |
| −7.27 | 1.52 |  | 2.52 | - | 3 | 8.14 | 0.28 |
| −5.05 | 3.74 |  | - | - | 2 | 5.19 | 0.09 |
| −1.44 | 7.35 |  | - | −0.22 | 3 | 5.22 | 0.02 |
